# Adenovirus Tales: From the Cell Surface to the Nuclear Pore Complex

**DOI:** 10.1371/journal.ppat.1004821

**Published:** 2015-06-04

**Authors:** Eric J. Kremer, Glen R. Nemerow

**Affiliations:** 1 Institut de Génétique Moléculaire de Montpellier, CNRS, Montpellier, France; 2 Université de Montpellier, Montpellier, France; 3 Department of Immunology and Microbial Science, The Scripps Research Institute, La Jolla, California, United States of America; University of Michigan Medical School, UNITED STATES

## Introduction

**Fig 1:**
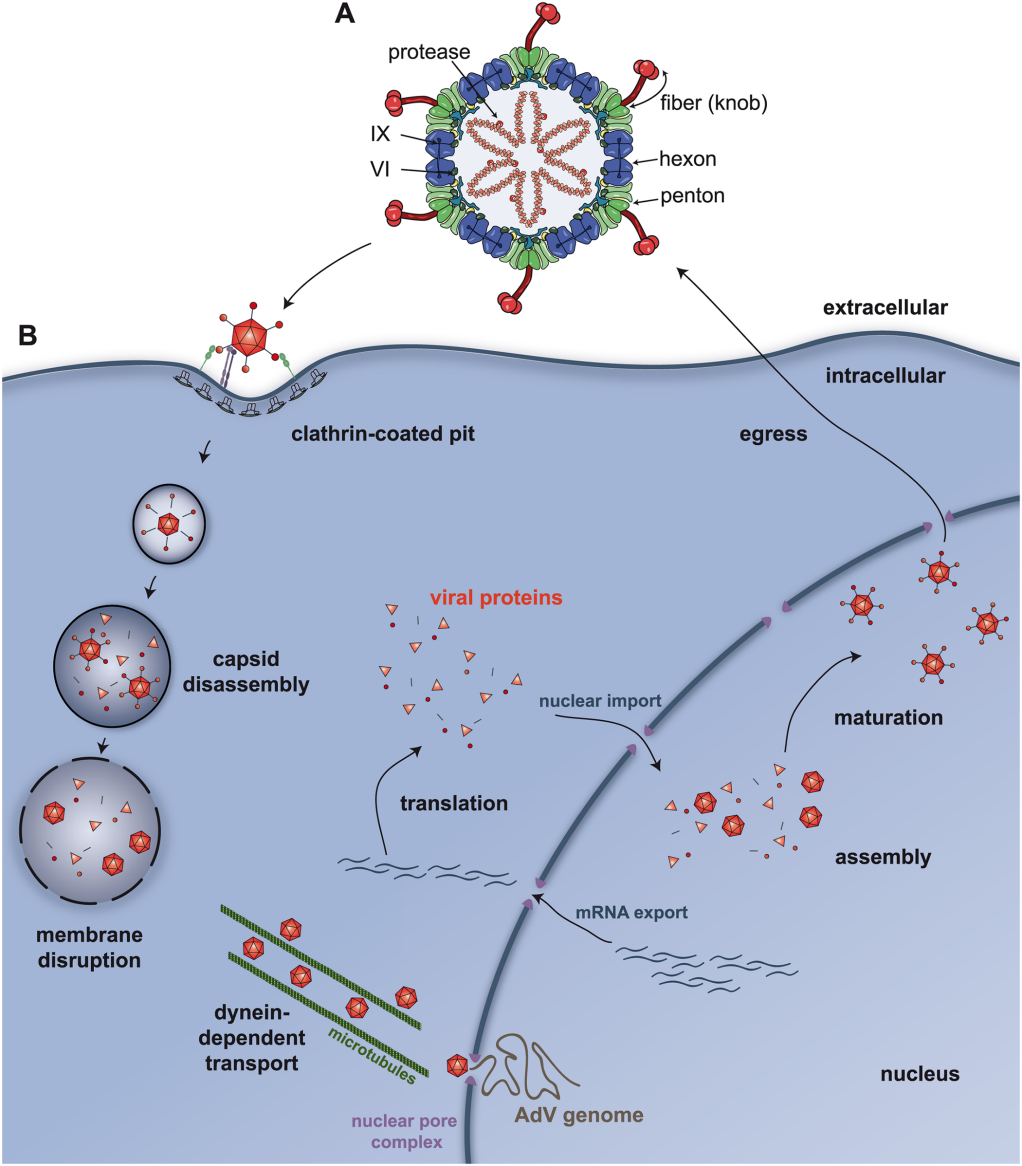
Adenovirus structure and trafficking. A) An illustration of the cross-section of a prototype 90 nm AdV capsid showing the location of the principal capsid proteins (hexon, penton, protein VI, protein IX, protease, and the fibre—the knob is the globular head of the fibre) involved in trafficking. B) An illustration showing the quintessential steps of AdV trafficking in epithelial cells. Via the knob region of the fiber, the capsid engages the cellular receptor. In some cell types, fibres are lost from the metastable* capsid during internalization in clathrin-coated pits. Postinternalization, the capsid continues to dissociate and releases protein VI, which allows the capsid access to the cytosol and interaction with dynein, then dynein-dependent transport along microtubule to the nuclear pore complex. *Metastable is a common term used to describe the biophysical state of fully mature nonenveloped virions. Overall, the particle is stable to the environment; however, it is able to respond to cellular cues to undergo conformational changes during cell entry.

Despite lingering safety concerns [1] and potential restrictions imposed by the host immune response, including the innate immune pattern recognition receptors (PRR), replication-defective and conditionally replicating human and nonhuman adenovirus (AdV) vectors continue to be a favorite vehicle for short-term (e.g., vaccine) and long-term gene delivery. This is due in part to several desirable features of AdV including their broad tissue tropism, their ample capacity for foreign gene insertion, and their LEGO-like structural adaptability (Fig 1A) to add, delete, and swap proteins and motifs from other viruses or host molecules.

While much is known about the molecular genetics and replication of AdVs, many investigators are continuing to decipher the captivating intracellular events of the first thirty minutes in the virus life cycle. This Pearl accentuates the strikingly diverse mechanisms for AdV entry, comparing human AdV type 5 (HAdV-C5) in epithelial cells and canine type 2 (CAdV2, or commonly referred to as CAV-2) in neurons. Similar viral and cellular proteins are used, and although the function of the cellular protein varies among cell types, these cell protein—virus associations promote similar outcomes. We also highlight some outstanding questions and hurdles needed to improve vector-mediated gene and vaccine delivery and treatments for AdV disease. The take home message is that one may be able to take advantage of a better understanding of these cell entry variations to control AdV pathogenesis and vector tropism for gene therapy.

## Mi Casa Es Su Casa: The Cellular Determinants That Dictate AdV Tropism

Of the more than 60 human AdV (HAdV) types that make up the current seven species (denoted as A–G), the most extensively studied are species C type 2 (HAdV-C2) and HAdV-C5. Many human and some nonhuman AdVs, including CAV-2, use the coxsackievirus and adenovirus receptor (CAR) [2–4] for high affinity attachment to host cells via the capsid fibre protein. On polarized epithelial cells, the predominant CAR isoform is targeted to the basolateral surface and in tight junctions. A minor exon 8-containing CAR isoform may be targeted to the apical surface [5] of some epithelial cells and allow easier access of CAR-tropic virus attachment. Other HAdV types from species B use desmoglein 2 or CD46, a member of the complement regulatory protein family, while species D HAdVs appear to use CAR, sialic acid, the GD1a glycan, and/or CD46 (for a recent review on AdV receptors see [6]). For HAdVs that use CAR as an attachment molecule on epithelial cells, engagement of the αv integrin is needed for efficient internalization (Fig 1B). This engagement occurs through association of the integrin with a consensus integrin interacting motif (RGD in most AdVs) located on an extended loop on the penton base [7]. Integrin ligation triggers signaling events that promote virus entry into early endosomes via clathrin-mediated endocytosis (Fig 1B). In epithelial cells, it seems that CAR facilitates attachment but not cell entry [8]. However, it is still unclear how significantly the integrin repertoire involved in membrane penetration influences different AdV types. Moreover, when injected intravenously in mice, some AdVs can interact with specific coagulation factors [9] that alter tissue tropism by preventing binding of naturally occurring antibodies and then by acting as a bridge to attach to proteoglycans on liver cells [10]. That coagulation factors influence HAdV tropism in rodents is clear, but its relevance for HAdV disease and HAdV vector administration in humans is unknown. As discussed below, AdV trafficking into neurons follows a pathway different from that of epithelial cells. Thus, the routes and modes of AdV cell entry are variable and cell-type dependent.

## Houston, We Have a Problem: The Escape Route HAdV Uses to Reach the Nucleus

Internalization of AdV particles is a primordial event for infection—but it is only the beginning of the journey to the nuclear pore complex (NPC). The ligation of CAR and integrins on the cell surface induces distinct membrane trafficking processes that produce a mechanical force to initiate partial capsid disassembly [11]. Analyses using atomic force microscopy are consistent with this model and indicate that integrin ligation by the virus is sufficient to loosen the vertex region(s) of the capsid [12]. Once inside most cells, the “metastable” virions ultimately need to escape a vesicular compartment to be translocated via a dynein-dependent mechanism to the NPC (Fig 1B). Removal of the vertex region—composed of the penton base, fibre [13], and likely the peripentonal hexons—allows release of the membrane lytic protein VI from the inner surface of the virus capsid [14]. Interestingly, α defensins HD5 and HNP1 bind to the vertex region of the virus capsid and prevent its disassembly. This restricts release of protein VI and membrane destruction. Exposure of the inner core of the virus apparently starts in the endosome as monitored by antibody detection of the viral genome at early time points [15]. Protein VI release is associated with increased endosomal membrane destruction [13], and a single point mutation (L40Q) in the amphipathic helical domain of protein VI significantly attenuates membrane insertion, membrane destruction, and cell infection [16]. Protein VI insertion into a lipid bilayer causes positive membrane curvature [17], and this may impart stress and global membrane destruction allowing passage of the partially disassembled capsid into the cytosol. If there are structural rearrangements that occur in protein VI after its release from the HAdV capsid, these could be druggable targets.

Partially uncoated virions also escape endocytic vesicle concomitant with a drop in pH. Yet, the precise role of pH in virus disassembly and/or engagement of molecular motors is enigmatic [18]. Partially uncoated virions can associate with dynein motor proteins that recognize the hexon [19] or possibly protein VI [20]. This association is instrumental in transporting the virions along microtubules, and numerous laboratories have seen that, in superinfected cells, AdV capsids can accumulate at the microtubule-organizing center. However, it is unclear whether the microtubule-organizing center is a launching pad for NPC engagement, an artifact of superinfected cells, or a cul-de-sac. The association of the virion with proteins at the NPC likely facilitates further uncoating of the virion, thereby allowing the genome to be translocated into the nucleus, although the precise mechanisms involved are still being investigated. Afterwards, the AdV genome is delivered to the nucleus to initiate a new round of propagation.

Whether capsid disassembly is initiated at a unique or specific vertex—for example, a “portal vertex” where the DNA is inserted during particle maturation—is unknown. Could a unique portal vertex be used for initial disassembly or protein VI release and nuclear import during infection and DNA packaging? If a single vertex pops off from the disassembled HAdV-C5 capsids, how would the genome become available to detection by cytosolic PRRs in some cell types? Clearly, cell type-specific trafficking influences the interaction with PRRs and therefore may provide an opportunity for vector optimization.

## The Penthouse Please: How Does CAV-2 Enter Neurons?

We all know that cells differ in their morphology and functions, and it would be surprising if they interacted with pathogens in identical manners. A distinct variation on AdV trafficking has been observed in neurons. CAV-2 has found an unlikely niche as a vector for gene transfer to central (brain and spinal cord) and peripheral (e.g., motor and sensory) neurons [21–23]. The basis of CAV-2’s preferential targeting to neurons is likely due to the exclusive use of CAR as both the attachment and entry molecule, as well as the selective expression of CAR on neurons in the brain parenchyma (versus microglia, astrocytes, and oligodendrocytes) and at neuromuscular junctions [24]. In addition to the preferential neuronal tropism, CAV-2 vectors are efficiently taken up at axon termini and transported back to the soma via retrograde transport [24,25]. While numerous studies of AdV trafficking in epithelial-like cells (including CAV-2 [26]) have led to a well-recognized pathway, CAV-2 trafficking in neurons accentuates AdV trafficking adaptability.

**Fig 2:**
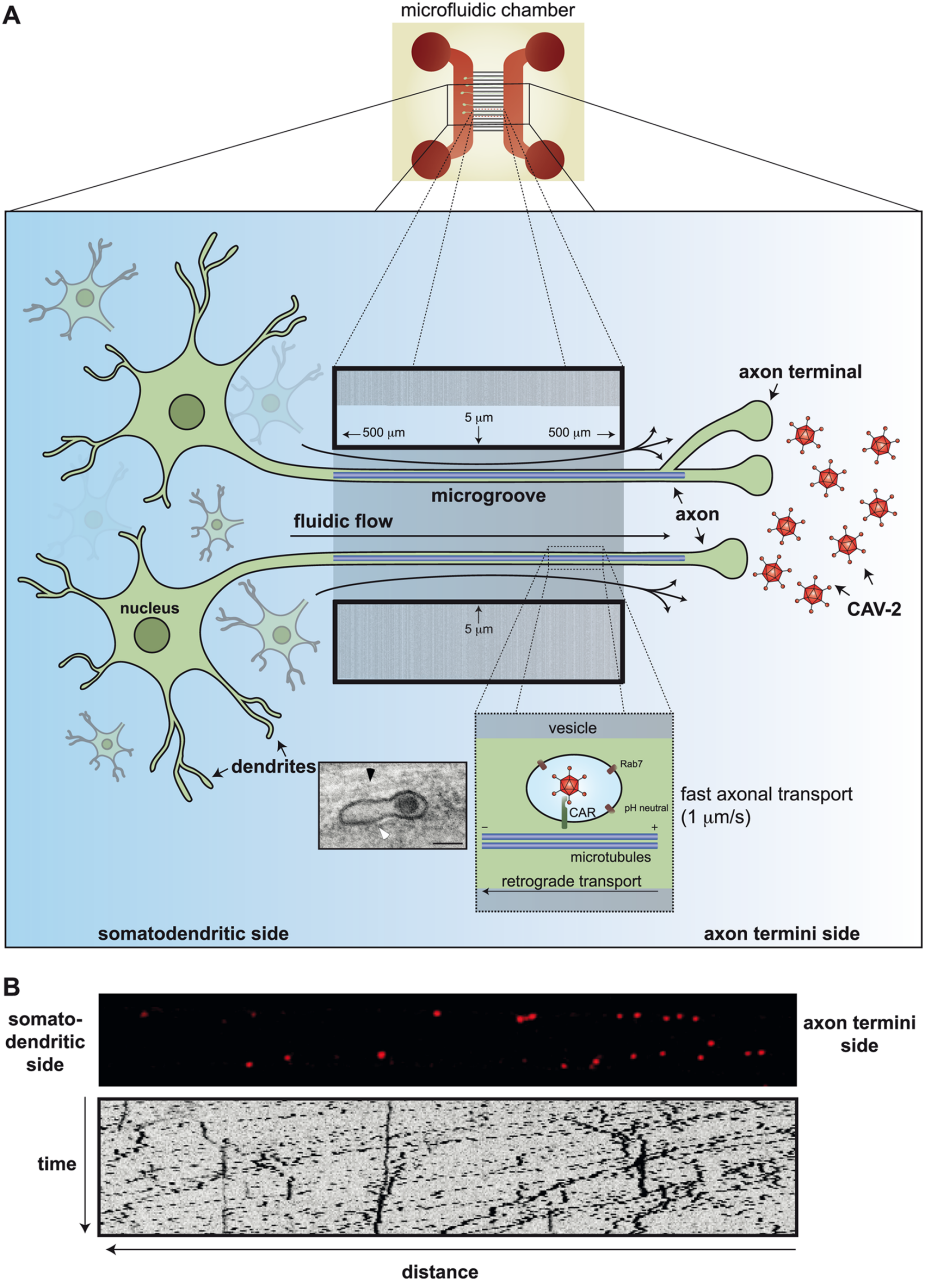
Axonal transport of CAV-2 in neurons. A) A schema showing the assays used to record CAV-2 directionality and speed in murine dorsal root ganglion (DRG) neurons. Neurons are cultivated in microfluidic chambers (top center) in which the microfluidic flow is from left to right. This flow of the medium in the 5-micron-wide and 500-micron-long microgroove prevents diffusion of particles and allows a physical separation between cell bodies (left) and axon termini (right). CAV-2, covalently labeled with a fluorophore (Cy3), were added for 90 min in the axonal compartment before the video was started, and axons in the middle of the microgroove were imaged at one frame/second. The rate of retrograde transport of CAV-2 in these conditions is approximately 1–2 microns/s (insert: ultrastructural electron micrograph of CAV-2 vesicular transport in motor neurons from Salinas et al. [31]). CAV-2 was mainly present in vesicular structures (white arrow) near microtubule tracks (black arrow). B) Still images of a microgroove of the chambers containing Cy3-labeled CAV-2 (red puncta) 90 min postincubation on the axon termini side. Below is a kymograph, which gives a graphical representation of the spatial position over time, of the corresponding movie (S1 Video). Scale bars in the micrograph = 100 nm.

CAV-2, like the two members of HAdV species F, does not contain an identifiable integrin-interacting motif in the penton base [27]. CAV-2 uses its trimeric fibre knob at the end of a double-hinged shaft [28] to attach to CAR with very high (1 nM) affinity. At axon termini, CAR is located in lipid rafts [29], and CAR-mediated internalization of CAV-2 appears to be induced by the disruption of intracellular homodimeric CAR interactions [29]. The number of CAR molecules needed to induce internalization is unknown, nor whether a torsional force is applied to the CAV-2 capsid during entry to induce the release of protein VI. It is also unclear whether neurons induce partial CAV-2 disassembly during internalization at, or near, the plasma membrane. The CAR-CAV-2 complex likely stays in lipid rafts, and internalization depends on actin reorganization, dynamin function, and—in contrast to HAdV-C5 internalization in epithelial cells—is clathrin-independent. Immediately postinternalization, the CAR-CAV-2 complex can be found in static Rab5^+^ vesicles that mature into Rab7^+^ vesicles, where active retrograde transport starts (Fig 2) [20].

The sorting platform for endocytosed vesicles is complex at the synapse: does CAV-2 influence the content of the vesicles it ends up in? Does this influence vesicular trafficking and targeting in neurons? And here is another difference between epithelial-like cells and neurons: CAV-2 does not escape from these CAR^+^/pH-neutral/Rab7^+^ multitasking compartments [30]. Of note, in epithelial cells, Rab7^+^ is a hallmark of late endosomes that have an acidic lumen. What appears to be an intact CAV-2 (Fig 2A insert) is transported to the soma of the neurons in a vesicular structure. This long, protected retrograde journey in motor neuron axons could be more than 1 meter in humans. When the CAR-CAV-2 complex reaches the soma, CAV-2 vesicular escape also coincides with a drop in the pH [31]. Whether the pH drop and endolysosome escape of CAV-2 in neurons are mechanistically linked has not been formally tested.

## Et Alors: The Implications for AdV-Mediated Gene Transfer

The modularity of the AdV capsid allows enormous adaptability for practical and theoretical vector design. With the more than 200 different AdVs partially or totally characterized to date, we have a rich reserve to create vectors for short- or long-term gene transfer. CAV-2 vector use in the brain is a perfect example [32]. Few would have predicted that CAV-2—which normally causes respiratory tract infection in some *Canidae* (e.g., dogs, wolves, foxes, bears, etc.)—would be a valuable tool with which to probe higher order brain function or as a vector to treat neurodegenerative diseases that affect the entire brain [33]. A CAV-2 vector eliminated the neuropathology in the mucopolysaccharidosis type VII (MPS VII) mouse and the MPS VII dog brain—a brain that is one-third the size of a two-year-old child. This therapeutic potential is due to the low immunogenicity, long-term transgene expression, ample cloning capacity [25], preferential transduction of neurons, and the use of the interconnectivity of neurons to reach structures throughout the brain. CAV-2 vector potential was unpredictable 20 years ago.

So, what are we waiting for? *Carpe momentum*.

## Supporting Information

S1 Video Mouse DRG neurons were cultivated in microfluidic chambers in which the microfluidic flow is from left to right. This fluidic flow of the medium in the 5-micron-wide and 500-micron-long microgroove prevents diffusion of particles and allows a physical separation between cell bodies (left) and axon termini (right). CAV-2 particles covalently labeled with a fluorophore (Cy3) were incubated for 90 min in the axonal compartment before the video was started, and axons in the middle of the microgroove were imaged at one frame/second x 100 seconds.
(MOV)

## References

[1] Perreau M, Pantaleo G, Kremer EJ. Activation of a dendritic cell-T cell axis by Ad5 immune complexes creates an improved environment for replication of HIV in T cells. J Exp Med. 2008 24;205(12):2717–25. PMID: ISI:000261295300006. 10.1084/jem.20081786 18981239 PMC2585831

[2] Bergelson JM, Cunningham JA, Droguett G, Kurt-Jones EA, Krithivas A, Hong JS, et al Isolation of a common receptor for Coxsackie B viruses and adenoviruses 2 and 5. Science. 1997;275(5304):1320–3 9036860 10.1126/science.275.5304.1320

[3] Roelvink PW, Mi Lee G, Einfeld DA, Kovesdi I, Wickham TJ. Identification of a conserved receptor-binding site on the fiber proteins of CAR-recognizing adenoviridae. Science. 1999;286(5444):1568–71 10567265 10.1126/science.286.5444.1568

[4] Seiradake E, Lortat-Jacob H, Billet O, Kremer EJ, Cusack S. Structural and mutational analysis of human Ad37 and canine adenovirus 2 fiber heads in complex with the D1 domain of coxsackie and adenovirus receptor. J Biol Chem. 2006 3;281(44):33704–16. 16923808 10.1074/jbc.M605316200

[5] Excoffon KJ, Gansemer ND, Mobily ME, Karp PH, Parekh KR, Zabner J. Isoform-specific regulation and localization of the coxsackie and adenovirus receptor in human airway epithelia. PLoS ONE. 2010;5(3):e9909 10.1371/journal.pone.0009909 20361046 PMC2845650

[6] Arnberg N. Adenovirus receptors: implications for targeting of viral vectors. Trends Pharmacol Sci. 2012 33(8):442–8. 10.1016/j.tips.2012.04.005 22621975

[7] Stewart PL, Chiu CY, Huang S, Muir T, Zhao Y, Chait B, et al Cryo-EM visualization of an exposed RGD epitope on adenovirus that escapes antibody neutralization. EMBO J. 1997;16(6):1189–98. 9135136 10.1093/emboj/16.6.1189 PMC1169718

[8] Wickham TJ, Mathias P, Cheresh DA, Nemerow GR. Integrins alpha v beta 3 and alpha v beta 5 promote adenovirus internalization but not virus attachment. Cell. 1993;73(2):309–19. 8477447 10.1016/0092-8674(93)90231-e

[9] Waddington SN, McVey JH, Bhella D, Parker AL, Barker K, Atoda H, et al Adenovirus serotype 5 hexon mediates liver gene transfer. Cell. 2008 8;132(3):397–409. 10.1016/j.cell.2008.01.016 18267072

[10] Xu Z, Qiu Q, Tian J, Smith JS, Conenello GM, Morita T, et al Coagulation factor X shields adenovirus type 5 from attack by natural antibodies and complement. Nat Med. 2013 19(4):452–7. 10.1038/nm.3107 23524342

[11] Burckhardt CJ, Suomalainen M, Schoenenberger P, Boucke K, Hemmi S, Greber UF. Drifting motions of the adenovirus receptor CAR and immobile integrins initiate virus uncoating and membrane lytic protein exposure. Cell Host Microbe. 2011 18;10(2):105–17. 10.1016/j.chom.2011.07.006 21843868

[12] Snijder J, Reddy VS, May ER, Roos WH, Nemerow GR, Wuite GJ. Integrin and defensin modulate the mechanical properties of adenovirus. J Virol. 2013 3;87(5):2756–66. 10.1128/JVI.02516-12 23269786 PMC3571403

[13] Wiethoff CM, Wodrich H, Gerace L, Nemerow GR. Adenovirus protein VI mediates membrane disruption following capsid disassembly. J Virol. 2005 79(4):1992–2000. . 15681401 10.1128/JVI.79.4.1992-2000.2005 PMC546575

[14] Smith JG, Silvestry M, Lindert S, Lu W, Nemerow GR, Stewart PL. Insight into the mechanisms of adenovirus capsid disassembly from studies of defensin neutralization. PLoS Pathog. 2010;6(6):e1000959 10.1371/journal.ppat.1000959 20585634 PMC2891831

[15] Nguyen EK, Nemerow GR, Smith JG. Direct evidence from single-cell analysis that human {alpha}-defensins block adenovirus uncoating to neutralize infection. J Virol. 2010 84(8):4041–9. 10.1128/JVI.02471-09 20130047 PMC2849482

[16] Moyer CL, Wiethoff CM, Maier O, Smith JG, Nemerow GR. Functional genetic and biophysical analyses of membrane disruption by human adenovirus. J Virol. 2011 85(6):2631–41. 10.1128/JVI.02321-10 21209115 PMC3067937

[17] Maier O, Galan DL, Wodrich H, Wiethoff CM. An N-terminal domain of adenovirus protein VI fragments membranes by inducing positive membrane curvature. Virology. 2010 20;402(1):11–9. 10.1016/j.virol.2010.03.043 20409568 PMC3028504

[18] Suomalainen M, Luisoni S, Boucke K, Bianchi S, Engel DA, Greber UF. A direct and versatile assay measuring membrane penetration of adenovirus in single cells. J Virol. 2013 87(22):12367–79. 10.1128/JVI.01833-13 24027314 PMC3807902

[19] Bremner KH, Scherer J, Yi J, Vershinin M, Gross SP, Vallee RB. Adenovirus transport via direct interaction of cytoplasmic dynein with the viral capsid hexon subunit. Cell Host Microbe. 2009 17;6(6):523–35. 10.1016/j.chom.2009.11.006 20006841 PMC2810746

[20] Wodrich H, Henaff D, Jammart B, Segura-Morales C, Seelmeir S, Coux O, et al A capsid-encoded PPxY-motif facilitates adenovirus entry. PLoS Pathog. 2010 6(3):e1000808 10.1371/journal.ppat.1000808 20333243 PMC2841620

[21] Hnasko TS, Perez FA, Scouras AD, Stoll EA, Gale SD, Luquet S, et al Cre recombinase-mediated restoration of nigrostriatal dopamine in dopamine-deficient mice reverses hypophagia and bradykinesia. Proc Natl Acad Sci U S A. 2006 6;103(23):8858–63. . 16723393 10.1073/pnas.0603081103 PMC1466546

[22] Pivetta C, Esposito MS, Sigrist M, Arber S. Motor-Circuit Communication Matrix from Spinal Cord to Brainstem Neurons Revealed by Developmental Origin. Cell. 2014 30;156(3):537–48. PMID: ISI:000330580800019 10.1016/j.cell.2013.12.014 24485459

[23] Ekstrand MI, Nectow AR, Knight ZA, Latcha KN, Pomeranz LE, Friedman JM. Molecular profiling of neurons based on connectivity. Cell. 2014 22;157(5):1230–42. 10.1016/j.cell.2014.03.059 24855954 PMC4854627

[24] Soudais C, Laplace-Builhe C, Kissa K, Kremer EJ. Preferential transduction of neurons by canine adenovirus vectors and their efficient retrograde transport in vivo. FASEB J. 2001;15(12):2283–5. 11511531 10.1096/fj.01-0321fje

[25] Soudais C, Skander N, Kremer EJ. Long-term in vivo transduction of neurons throughout the rat CNS using novel helper-dependent CAV-2 vectors. FASEB J. 2004 18(2):391–3. 14688208 10.1096/fj.03-0438fje

[26] Chillon M, Kremer EJ. Trafficking and propagation of canine adenovirus vectors lacking a known integrin-interacting motif. Hum Gene Ther. 2001 20;12(14):1815–23. 11560774 10.1089/104303401750476302

[27] Soudais C, Boutin S, Hong SS, Chillon M, Danos O, Bergelson JM, et al Canine adenovirus type 2 attachment and internalization: coxsackievirus-adenovirus receptor, alternative receptors, and an RGD-independent pathway. J Virol. 2000 74(22):10639–49. 11044108 10.1128/jvi.74.22.10639-10649.2000 PMC110938

[28] Schoehn G, El Bakkouri M, Fabry CM, Billet O, Estrozi LF, Le L, et al Three-dimensional structure of canine adenovirus serotype 2 capsid. J Virol. 2008 82(7):3192–203. 10.1128/JVI.02393-07 18216088 PMC2268473

[29] Salinas S, Zussy C, Loustalot F, Henaff D, Menendez G, Morton PE, et al Disruption of the coxsackievirus and adenovirus receptor-homodimeric interaction triggers lipid microdomain- and dynamin-dependent endocytosis and lysosomal targeting. J Biol Chem. 2014 10;289(2):680–95. 10.1074/jbc.M113.518365 24273169 PMC3887196

[30] Salinas S, Schiavo G, Kremer EJ. A hitchhiker's guide to the nervous system: the complex journey of viruses and toxins. Nat Rev Microbiol. 2010 8(9):645–55. PMID: ISI:000280855500011 10.1038/nrmicro2395 20706281

[31] Salinas S, Bilsland LG, Henaff D, Weston AE, Keriel A, Schiavo G, et al CAR-associated vesicular transport of an adenovirus in motor neuron axons. PLoS Pathog. 2009 5(5):e1000442 10.1371/journal.ppat.1000442 19461877 PMC2677547

[32] Bru T, Salinas S, Kremer EJ. An update on canine adenovirus type 2 and its vectors. Viruses. 2010 9;2(9):2134–53. 10.3390/v2092134 21994722 PMC3185752

[33] Cubizolle A, Serratrice N, Skander N, Colle MA, Ibanes S, Gennetier A, et al Corrective GUSB Transfer to the Canine MPS VII Brain. Mol Ther. 2014 22(4):762–73. PMID: ISI:000334061100010 10.1038/mt.2013.283 24343103 PMC3983960

